# Acetanilid as a Local Antiseptic and Analgesic

**Published:** 1896-04

**Authors:** Lucien Lofton

**Affiliations:** Atlanta, Ga.


### ACETANILID AS A LOCAL ANTISEPTIC AND ANALGESIC.

BY LUCIEN LOFTON, M. D., ATLANTA, GA.

The virtues of numberless local applications to replace iodoform have time and again been extolled, and in presenting a few clinical facts as to the efficiency of acetanilid, both as a local antiseptic and analgesic, I do so from the standpoint of practical experience.

It is useless almost to say that in the application of iodoform as a local dressing, excepting an occasional dermatitis and iodic acne, the principal disturbing element in this preparation is its odor, hence many suggestions and theories have been proffered for the elimination of this objectionable feature of the powder.

Acetanilid is of course recognized as one of the milder coal-tar derivatives, and as an antipyretic has been used for several years with varying benefit. But it is chiefly desired in this article that the drug should be reviewed as a local antiseptic and anodyne.

For the past two years at the Southern Medical College, I have used acetanilid in the treatment of chancroids, herpes, balanitis, gonorrhœa, scabies, scalds, burns, eczema and indolent ulcers, besides other abnormal local conditions, and externally as a powder in sundry flesh wounds where primary union was desired. The results following my labors have been most admirable. These experiments were made principally upon colored patients at the college. I designate the material experimented upon, from the fact of the enormous difficulties which present themselves in the treatment of the negro. In the first place you are almost handicapped, owing to the irresponsibility, and irregularity of the patient in attending, while secondly, the various dyscrasiæ that invariably exist in this degenerate and profligate race, play no minor role in the battle for good nutrition to the seat of lesion. I have treated all manner of recent skin lesions with acetanilid and have never had a single instance of suppuration.

In chronic dermal diseases the drug was applied as an ointment, varying from ten to forty per cent. In old ulcers, where curetting, strapping, local tonics and stimulants had been persisted in, in some instances for months, the daily application of pulverized acetanilid proved effectual, many times arousing a sluggish sore to healthy granulations within forty-eight hours. In burns, scalds and chilblains, where the true skin was involved, the thorough dusting of tne injured part with the preparation allayed pain and made the patient comfortable in a few minutes.

I have used acetanilid both as a powder and ointment in burns and scalds covering large area, with entire satisfaction, and absolutely no toxic effect. In one instance I recall, the compound was applied as a powder upon a colored child four years of age, who had been scalded so severely that the entire anterior and lateral aspects of the lower extremities were denuded of their surfaces, without the slightest deleterious physiological effect. The patient's legs and thighs were dressed every third day, by sprinkling the parts thickly with pulverized acetanilid, and over this was placed soft sterilized cloths, besides cotton batting and a bandage loosely wound. From the effects of the injury the child made a happy recovery, and little, if any scarring, or distortion remains.

In the management of sores, proved by experiment to be a chancroidal, a definite recovery was secured within a week, by the application alone of the drug. In specific urethritis in the male, urethral suppositories made of cocoa butter containing one gramme of the remedy, was tried twice each day, while a twenty per cent. aqueous solution was injected three times each day, resulting in cures. Owing to the great insolubility of acetanilid, the solution is rather a mixture, and in consequence has to be thoroughly shaken when used. In an irritable urethra I know of nothing which acts as an analgesic with so much regularity and promptness as does acetanilid, either in the form of a bougie or an injection. Vaginitis of a specific origin may be treated with a douche, using from two to six grammes of acetanilid to two liters of water, as hot as can be borne. This may be repeated every three or four hours. Hyperidrosis of the feet may be greatly benefitted, and in some cases cured, by the continued application of the compound, dusting about one gramme in the sock or stocking upon rising. In all true skin diseases an ointment will be found more beneficial than a powder.

In making this plea for the use of acetanilid as a local application, I do not mean to imply that it is a cure-all, but in the hands of a careful and painstaking observer, its numerous virtues will inevitably be demonstrated. The drug is cheap, and therefore economical. I have only mentioned a few instances in which acetanilid may be utilized, but if its remedial properties be as lasting and faithful in the future as they have been in the past with me, I shall remain its unconditional champion.

